# Integration of Patient-reported Outcomes in a Total Joint Arthroplasty Program at a High-volume Academic Medical Center

**DOI:** 10.5435/JAAOSGlobal-D-20-00034

**Published:** 2020-05-01

**Authors:** Surabhi Bhatt, Kristina Davis, David W. Manning, Cynthia Barnard, Terrance D. Peabody, Nan E. Rothrock

**Affiliations:** From the Department of Orthopaedic Surgery (Ms. Bhatt, Dr. Manning, Dr. Peabody), Northwestern University Feinberg School of Medicine; the Northwestern Memorial HealthCare (Ms. Davis, Dr. Barnard), Northwestern University Center for Healthcare Studies; the Department of Medical Social Sciences (Dr. Rothrock), Northwestern University Feinberg School of Medicine; and the Department of Surgery (Dr. Barnard), Feinberg School of Medicine, Northwestern University, Chicago, IL.

## Abstract

**Introduction:**

Despite widely appreciated barriers to successful clinical implementation, the literature regarding how to operationalize electronic health record-integrated patient-reported outcomes (PROs) remains sparse. We offer a detailed summary of the implementation of PROs into the standard of care at a major tertiary academic medical center.

**Methods:**

Collection of four Patient-Reported Outcomes Measurement Information System computer adaptive tests was piloted in a large academic orthopaedic surgery ambulatory clinic starting in October 2016. The Patient-Reported Outcomes Measurement Information System computer adaptive tests (Physical Function, Pain Intensity, Pain Interference, and Ability to Return to Social Roles and Activities) were initially implemented as manual order sets to be administered before surgery through 2 years after surgery. Completion rate over time, mean time to completion for all PRO domains, and the overall distribution of symptom severity were used to evaluate the success of the pilot. A subsequent optimization and redesign of the pilot was conducted using tablets, automation of questionnaire deployment, and improved results review to address obstacles encountered during the pilot phase.

**Results:**

Two thousand nine distinct joint arthroplasty patients (mean age = 65) completed at least one set of PRO assessments, with overall completion rates reaching 68% and mean completion time of 3 minutes. Focal points during the implementation process included engagement and training of staff, selection of an appropriate patient population and outcome measures, and user friendly data displays for patients and providers.

**Conclusion:**

Our pilot program successfully demonstrated that PROs can be administered, scored, and made immediately available within the electronic health record to patients and their providers with minimal disruption of clinical workflows. Although considerable operational and technological challenges remain, we found that the implementation of PROs in clinical care within an ambulatory practice at an academic medical center can be achieved through a constellation of several key factors.

Health care in the United States is continuing to evolve toward a value-based system in which compensation is increasingly driven by patient-centered clinical outcomes.^1^ This requires defining clinical value and justifying costs of clinical interventions, particularly in areas such as orthopaedic surgery. Although traditional clinician-mediated measures of patient symptoms and outcomes such as clinical examination and radiographic results provide valuable information, they do not directly measure a patient's concerns and symptom burden. Patient-reported outcomes (PROs) are “any report of the status of a patient's health condition that comes directly from the patient, without interpretation of the patient's response by a clinician or anyone else.”^2^ Broadly defined, they quantify health-related quality of life such as health perceptions, well-being, symptoms, and function. PRO measures can assess universal (eg, fatigue or physical function) or disease-specific (eg, osteoarthritis of the knee) symptoms or function. A wide array of PROs have been validated for use in clinical research after surgical interventions and in many chronic and acute conditions such as depression, cancer, chronic obstructive pulmonary disease, and heart failure.^3,4,5,6,7,8,9,10^ PROs can capture patients' perspectives on the severity and impact of symptoms on physical health and mental well-being. Routine inclusion of PROs in care has the potential to improve treatment decision-making, focus patient-provider discussions on the most burdensome symptoms, and increase patients' engagement in their care.^11,12,13,14,15,16^ PROs may also be better measures of the targets of care and therefore useful in evaluating the healthcare quality rather than relying solely on provider-centric metrics.^17^

Determining how to collect and efficiently use PRO data to improve the health of patients continues to present a challenge for health systems. Although multiple studies have offered guidance on methodological and practical decisions to implement PROs in practice,^18,19,20,21^ few teams have described the development of a PRO program implemented in a high-volume setting that can be potentially applicable across multiple clinics and conditions.^22-24^

The Northwestern Medicine Patient-Reported Outcomes (NMPROs) platform was developed as an integrated tool within the electronic health record (EHR) system Epic (Madison, WI) used at our institution to administer, score, and display Patient-Reported Outcomes Measurement Information System computer adaptive tests through the patient communication portal in Epic MyChart at home or in a clinic at the point of care. This study outlines the pilot implementation of PROs in the Total Joint Replacement Program in the Department of Orthopaedic Surgery as a model for other large and complex high-volume academic healthcare systems.

## Methods

### System Design Features

The most critical decision made in the development of NMPRO was to fully integrate it within the EHR. This integration was chosen primarily because it enables clinician access to PRO scores in the same location as all other health information, thereby facilitating the use of the data clinically in care in addition to making the data available through the enterprise data warehouse for future retrospective research activities. The Northwestern Medicine Enterprise Data Warehouse is a single, comprehensive and integrated repository of all clinical and research data sources to facilitate research, clinical quality, healthcare operations, and medical education at our institution. The NMPRO design also includes the ability to select clinically relevant PROs for each population of interest. Administration is flexible and can be triggered by condition-specific, time-based triggers or through an on-demand feature at the clinician's discretion with the ability for patients to complete the assessments at home or in clinic. Finally, NMPRO provides the clinical staff with immediate access to the results within the EHR.

### Governance

A steering committee consisting of a multidisciplinary group of stakeholders was established to provide guidance and oversight for coordination of PRO implementation. They serve as a governing body and prioritize expansion efforts across the healthcare system.

### Selection of Pilot Clinic

The Total Joint Replacement Program in the Department of Orthopaedic Surgery was chosen as a pilot for three primary reasons. First, there was considerable interest from the surgical team to monitor changes in symptom severity over time, support shared clinical decision-making, assess treatment effectiveness, and use the data generated for future research endeavors. Second, the Centers for Medicare and Medicaid Services expressed interest in using PROs in the future performance measures for value-based purchasing and satisfying meaningful use requirements.^25^ Finally, the program had a high level of readiness and high levels of engagement from key operational partners.

### Key Features of Implementation

A four-step approach of prioritization and resource planning was applied to implement PROs in Orthopaedic Surgery:

(1) Preimplementation phase—identification of key operational and clinical partners, assessment of readiness and scope of change needed to current-state workflow, and selection of appropriate PRO measures per clinical requirements.
(2) Technical build phase—technical Epic build programming, functionality testing, and technical build revisions.
(3) Implementation phase—education/training of clinical staff, live demonstrations, and at-the-elbow support.
(4) Postimplementation phase—formal postimplementation review, analysis of postimplementation data, and development of optimization plan.

### Pilot Design

The orthopaedic surgery pilot included all English-speaking patients undergoing primary and revision total hip and knee arthroplasties. Non-English speakers were excluded from the pilot to remove possible confounding effects such as language barriers and health literacy issues. PROs were collected using three manual order sets (presurgery, postsurgery, and on-demand) built to assess the following validated Patient-Reported Outcomes Measurement Information System computer adaptive test domains relevant to orthopaedic surgery patient populations: Physical Function, Pain Interference, Pain Intensity, and Ability to Participate in Social Roles and Activities. Each domain poses questions to patients regarding their level of physical difficulty or severity of pain in completing a variety of daily activities, such as household chores or socialization with friends and family. The assessment duration was kept to a maximum of 8 minutes to reduce thev risk of fatigue and potential abandonment.

**Figure 1:**
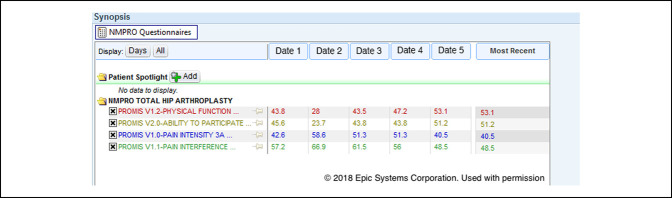
Photograph showing the electronic health record patient-reported outcome results display—longitudinal scores.

**Figure 2:**
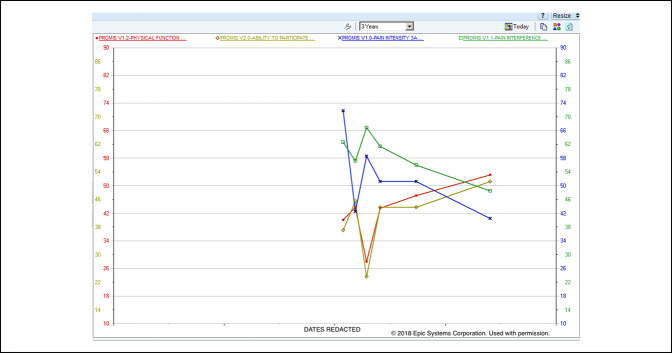
Graph showing the electronic health record patient-reported outcome results display—longitudinal graph.

Customized workflows were developed for each surgical team to place the presurgical and postsurgical orders at appropriate points in care. The presurgical PRO assessments were completed during the presurgical visit in the clinic using the examination room workstations. The postsurgery order set was placed in Epic the week before surgery (“P1”) by a member of the surgeon's clinical team to trigger an automatic cascade of five time windows chosen by the surgeons based on meaningful clinical benchmarks for recovery over time: 3 weeks (±2 weeks, “Post 1”), 5 weeks (±2 weeks, “Post 2”), 10 weeks (±4 weeks, “Post 3”), 23 weeks (±4 weeks, “Post 4”), 1 year (±2 months, “Post 5”), and 2 years (±2 months, “Post 6”). These PRO assessments could be completed outside of the clinic through the patient portal or in the clinic during routine visits using the examination room workstations. The designated staff member in each surgeon's clinic reviewed the clinic schedule ahead of time to identify patients who had failed to complete the PRO assessments in advance using the MyChart portal and therefore needed to complete these assessments in the clinic. Once the PRO assessments were launched by the designated staff member in the clinic through a hyperlink window, the EHR was securely locked in the background. The designated staff member also remained available to answer any questions or clarify responses for the patient. The on-demand order set supplemented the presurgery and postsurgery order sets by initiating an additional PRO assessment during the course of care if required by the clinical team. Once completed, all PRO results were available in table and graphed formats for immediate review in the EHR (Figures 1 and 2).

Multiple training tools and procedures were also developed to aid with the implementation. “At-the-elbow” training was provided throughout the implementation phase to ensure staff were aware of the best practices in implementation and ways to integrate PROs into their workflow. In-person tutorials and user guides were also developed to aid with the determination of completion status and launching the PRO assessments. Scripts were developed for all clinic staff to promote standardization of all communication regarding the purpose and importance for PROs. Patient-facing educational handouts were developed to be included in all presurgical information packets, outlining the purpose of PROs.

Throughout the pilot period, the NMPRO implementation team members received qualitative feedback from providers, operational staff, and patients. Areas of confusion and frustration were prioritized as targets for process improvement in the subsequent optimization phase.

### Evaluation Metrics

Three metrics were collected to evaluate the success of the implementation in orthopaedic surgery and identify the areas for process improvement: completion rate, completion time, and PRO severity score distribution (Table 1).

**Table 1:** Evaluation Metrics

Category	Metric	Application
Completion rate	Percentage of patients who complete an assessment by month and by time point	Degree of success altering routine care to include PRO collection
Completion time	Time to complete one assessment	Burden for patients and clinic workflow
PRO score distribution	Frequency of PRO measure scores by month and by assessment time point	Evaluation symptom severity experienced by sample and change over time and identification of potential clinician burden

PRO = patient-reported outcome

## Results

**Figure 3:**
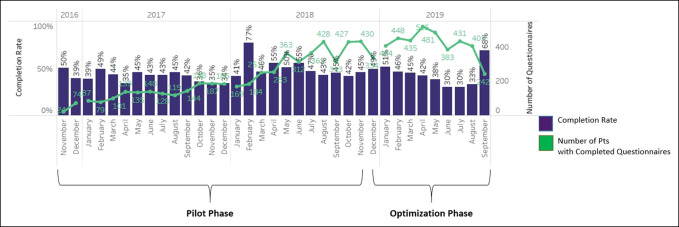
Graph showing the completion rates by month.

The results discussed herein consist of two phases: the initial pilot phase and the subsequent optimization phase. The pilot phase was defined as the period between October 2016 and December 2018. Four joint arthroplasty surgeons participated in the Orthopaedic Surgery pilot, performing total joint arthroplasty on 2,442 distinct patients. Of the patients who underwent a total joint arthroplasty during the pilot phase, 2,004 distinct patients (82%) had PRO assessment orders placed using manual order sets. Of these, 1,151 (57%) (mean age 66 years, range 17 to 95 years) completed at least one set of PRO assessments. Presurgical and postsurgical PRO assessment completion peaked at 77% between October 2016 and December 2018 and demonstrated notable variation within this period (range 34% to 77%; Figure 3).

Through qualitative feedback, clinical and operational staff identified multiple challenging aspects of the pilot implementation including (1) integrating the collection of PROs seamlessly into the patient encounter through integration into workflows, (2) getting the correct PRO triggered to the correct patient at the correct time, and (3) engaging and educating the patient to promote PRO completion. Consequently, after the pilot phase, the NMPRO implementation team initiated an optimization phase, defined as the period between December 2018 and September 2019 in which the team deployed strategies aimed at addressing the obstacles affecting evaluation metrics as discussed in detail below. During this period, the four orthopaedic surgeons performed a total joint arthroplasty on 997 distinct patients. Of the patients who underwent a total joint arthroplasty, 858 distinct patients (86%) (mean age 65 years, range 17 to 100 years) had PRO assessment generated and 229 (26%) completed at least one set of PRO assessments. The discrepancy between surgical case volume and PRO assessments generated was attributable to patients who underwent bilateral surgeries and were not prompted to begin a new assessment series. The rationale for this decision was that patients would not be able to accurately distinguish symptoms and physical limitations after one procedure versus the other. Finally, presurgical and postsurgical PRO assessment completion reached 68% between December 2018 and September 2019 and demonstrated notable variation over time (range 30% to 68%; Figure 3).

### Optimization Phase

#### Use of Tablets to Expedite Collection

The pilot initially involved PRO completion using the workstations in clinic examination rooms, but the required time to complete the PRO assessments impeded the workflow of medical assistants and nurses to complete their evaluations. Tablets were consequently introduced in August 2018 to test alternative ways to collect measures during routine downtime during a patient's clinic visit (eg, after check-in in the waiting area). Although the use of tablets increased the number of staff who could initiate an assessment, no notable change was observed in the overall completion rate after their introduction (Figure 3). The lack of improvement in completion rates was because of two primary issues, namely, the need for clinical staff to log into multiple internal systems to identify each patient with an active questionnaire and often needing to assist patients who were unfamiliar with completing the assessments on these devices.

#### Automation of Assessment Deployment

The initial implementation required manual order placement for the pre-op and post-op PRO assessment series for each patient. Consequently, the completion rates were dependent on clinic staff to place the correct order at the correct time. Some patients were therefore missed, enrolled late, or enrolled early. Automated questionnaire deployment using the current procedural terminology procedural codes to trigger both the presurgery and postsurgery PRO assessments was implemented in December 2018. Although more assessments were triggered and made available for completion, no actual change was observed in the completion rates between the pilot and optimization phase (Figure 3).

#### Patient Engagement through Results Review

Providing information on the purpose of PROs including their role in identifying care needs has been identified as critical for patient engagement.^26^ The surgeons attempted to review the results of the PROs with each patient during their clinic visit as an additional data point to patient's progress, describing the routine nature of PROs as similar to the ubiquity of radiographs in orthopaedic care. Patients cited multiple reasons as to why PROs had not been completed, with most citing a lack of understanding regarding their purpose. Additional training to providers and operational staff was therefore provided in May 2019. Scripts for staff explaining PRO collection were also revised to emphasize rationale. Although the short time frame of available data limits the strength of our interpretation, an increase in overall completion rates (46% to 68%) was observed between May 2019 and September 2019 and may be attributed to revisions in the training and scripts provided to the providers and operational staff.

**Figure 4:**
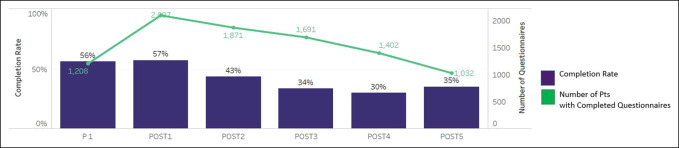
Graph showing the completion rates by time since surgery.

**Figure 5:**
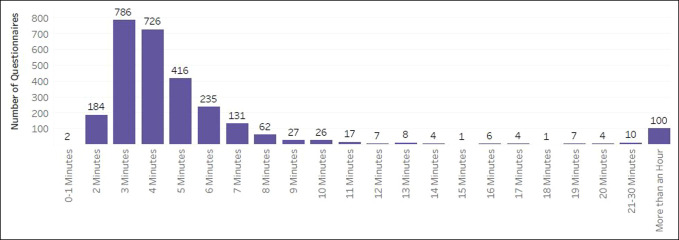
Graph showing the sssessment completion time (minutes).

**Figure 6:**
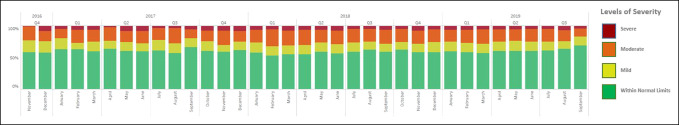
Graph showing the severity of symptoms by month.

**Figure 7:**
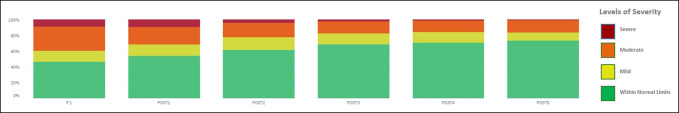
Graph showing the severity of symptoms before and after surgery.

**Figure 8:**
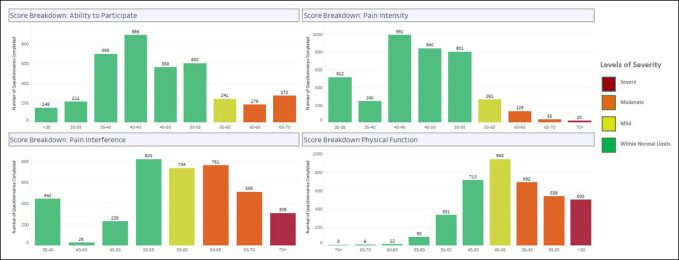
Graph showing the frequency distribution of patient-reported outcome measure scores.

Across both phases, the completion rates demonstrated attrition because time since surgery increased (Figure 4). The average length of time to complete the PRO assessments was 3 minutes (Figure 5). At the functional recovery level across all PRO measures and time points, the distribution of scores remained stable with over 50% of scores falling within the normal limits and only under 8% at the severe level (Figure 6). As expected, patients reported the worst symptoms and function presurgery and the scores improved over the first year (Figure 7). It is important to note that variance in severity at each postsurgical time point is confounded by attrition in the number of patients completing time points further out from surgery. For example, the observed increase in the proportion of responses indicating mild severity at postoperative time point six is counterintuitive to the assumption that patients continue to improve postoperatively. This trend is more likely to be because of a decreasing sample size of patients completing assessments at 24 months postoperatively, rather than a recurrence of symptoms after recovery through 12 months. Collapsing all time points together, score distributions ranged from over 2 SDs better and over 2 SDs worse than the US general population. Physical Function and Pain Interference had the highest number of scores in the severe and moderate range (Figure 8).

## Discussion

Overall, the integration of PRO measures into this busy ambulatory practice within an academic medical center was successful in many respects. Patients were observed to routinely complete the four PRO measures for physical function, pain, and social well-being in approximately 3 minutes on average. The scores demonstrated an expected range of severity, and the postsurgical improvement in these domains was well aligned with the surgeon's clinical expectations.

Despite the many successes of the NMPRO program, the implementation in Orthopaedic Surgery encountered multiple hurdles in the form of technological barriers, operational barriers, and overall challenges in quantifying the success of the implementation that are discussed in detail below.

### Technological Barriers

Multiple publications have documented the notable effort required to program customizable PROs into the EHR.^26-28^ Like others, we encountered similar hurdles that required notable resources, programming time, and management to address. The implementation required a designated information services analyst team member and software programmer who constructed the technical build of the PRO assessments and automated triggers within the EHR. The difficulty of integrating the assessment into the EHR was considerably more arduous than anticipated and will conceivably remain a potential area of difficulty for other systems. The information service team members therefore continued to be retained to perform functionality testing and troubleshoot a multitude of issues that materialized during the build. In addition, the implementation required oversight and management from a project manager to comprehensively support the rollout. Finally, the implementation required identifying specific operational leaders and clinical staff to install as dedicated team members tasked with operationalizing and managing the rollout within the practice.

Second, only approximately 66% of our patients currently use the patient communication portal MyChart to track their care and communicate with the care team. In NMPRO, MyChart is the primary mode for completing PRO assessments remotely. As a result, the completion rates declined because time since surgery increased. This decline in completion rates may be attributable to patients no longer returning for in-person follow-up, decreased symptom severity, or a variety of other reasons. Finally, the introduction of tablets to expedite the clinic workflow and improve PRO completion rates had no impact on the completion rates. Furthermore, the completed PRO results were not accessible to patients in an easily interpretable format to facilitate self-monitoring. Having the results accessible to the patients via MyChart could potentially have an effect on completion rates.

### Operational Barriers

Maintaining consistent engagement with process owners at key decision-making stages is fundamental to sustaining change in workflow and overall culture.^29^ Physician champions are vital in emphasizing the value of PROs and their utility in clinical care to their peers, role-modeling the use of PROs in the clinical encounter.^30,31^ However, despite concerted efforts, our department faced notable challenges in being able to effectively integrate the PROs into their workflow and convey their importance to patients.^32^ Lack of time and competing clinician priorities regularly interfered with review and discussion of PROs with patients. Additional training for patients and staff on the rationale for PRO collection did not alter the completion rates, suggesting that other interventions were needed. Although the automation of the PRO increased the number of assigned assessments, it proved insufficient in improving the completion rates. Embedding PROs as another “vital sign” similar to radiographs in a comprehensive view of patient heath status as previously mentioned will therefore require a cultural shift to view PRO data having the same foundational value as other clinical data.

### Challenges in Quantifying Success

Evaluation metrics are vital to advancing quality improvement, and research endeavors and insufficient PRO data volume greatly hampers clinicians' ability to use these data in a meaningful manner. Our primary evaluation metric, completion rate, varied over time. Furthermore, limited data prevent the construction of representative data sets that can be used to establish the norms for PRO scores over time. Consequently, a concerted effort to increase patient participation is vital. Evaluation of the frequency and efficacy of clinicians' use of PROs as a tool for patient education and engagement is needed.

The next phase of improvement in NMPRO will implement and assess modifications to increase PRO completion. These include the use of an electronic dashboard to track completion rates and encouraging point-of-care use. In addition, we are developing a patient friendly data visualization system accessible via the patient portal that could aid patients in tracking their outcomes over time. Engagement with clinician and patient stakeholders remains extremely important to achieve these goals.

## Conclusions

The pilot program successfully demonstrated that PROs can be administered, scored, and made immediately available within the EHR, with minimal disruption in the efficiency of clinical workflows. However, considerable operational and technological challenges remain. The NMPRO initiative offers a scalable model for process implementation and highlights key barriers to be considered during implementation. We assert that the NMPRO initiative can help guide PRO implementations in a variety of clinical populations and similarly structured hospitals and medical groups. The development of new strategies for more effective engagement and retention of patients to improve the response rates and accumulate a more complete longitudinal data set will continue to be vital in maximizing the utility and value of initiatives like ours.

## References

[1] Badash I Kleinman NP Barr S: Redefining health: The evolution of health ideas from antiquity to the era of value-based care. Cureus 2017;9:e1018. 28348937 10.7759/cureus.1018 PMC5346014

[2] Federal Drug Agency: Guidance for Industry: Patient-Reported Outcome Measures: Use in Medical Product Development to Support Labeling Claims. https://www.fda.gov/downloads/drugs/guidances/ucm193282.pdf. Accessed September 20, 2018. 10.1186/1477-7525-4-79 PMC1629006 17034633

[3] Cook KF Jensen SE Schalet BD: PROMIS^®^ measures of pain, fatigue, negative affect, physical Function and social function demonstrate clinical validity across a range of chronic conditions. J Clin Epidemiol 2016;73:89-102. 26952842 10.1016/j.jclinepi.2015.08.038 PMC5131708

[4] Marshall S Haywood K Fitzpatrick R: Impact of patient-reported outcome measures on routine practice: A structured review. J Eval Clin Pract 2006;12:559-568. 16987118 10.1111/j.1365-2753.2006.00650.x

[5] Hung M Clegg DO Greene T Saltzman CL: Evaluation of the PROMIS physical function item bank in orthopaedic patients. J Orthop Res 2011;29:947-953. 21437962 10.1002/jor.21308

[6] Jensen RE Potosky AL Reeve BB: Validation of the PROMIS physical function measures in a diverse US population-based cohort of cancer patients. Qual Life Res 2015;24:2333-2344. 25935353 10.1007/s11136-015-0992-9 PMC5079641

[7] Hung M Baumhauer JF Latt LD Saltzman CL SooHoo NF Hunt KJ; National Orthopaedic Foot & Ankle Outcomes Research Network: Validation of PROMIS^®^ physical function computerized adaptive tests for orthopaedic foot and ankle outcome research. Clin Orthop Relat Res 2013;471:3466-3474. 23749433 10.1007/s11999-013-3097-1 PMC3792246

[8] Flynn KE Dew MA Lin L: Reliability and construct validity of PROMIS measures for patients with heart failure who undergo heart transplant. Qual Life Res 2015;24:2591-2599. 26038213 10.1007/s11136-015-1010-y PMC4593724

[9] Amtmann D Kim J Chung H: Comparing CESD-10, PHQ-9, and PROMIS depression instruments in individuals with multiple sclerosis. Rehabil Psychol 2014;59:220-229. 24661030 10.1037/a0035919 PMC4059037

[10] Irwin DE Atwood CA Jr Hays RD: Correlation of PROMIS Scales and clinical measures among chronic obstructive pulmonary disease patients with and without exacerbations. Qual Life Res 2015;24:999-1009. 25307510 10.1007/s11136-014-0818-1 PMC4369165

[11] Baumhauer JF Bozic KJ Value-based healthcare: Patient-reported outcomes in clinical decision making. Clinorthoprelat Res 2016;474:1375-1378. 10.1007/s11999-016-4813-4 PMC4868147 27052020

[12] Hughes TM Merath K Chen Q: Association of shared decision-making on patient-reported health outcomes and healthcare utilization. A J Surg 2018;216:7-12. 10.1016/j.amjsurg.2018.01.011 29395026

[13] Freel J Bellon J Hanmer J Better Physician Ratings From Discussing PROs with Patients. https://catalyst.nejm.org/ratings-patients-discussing-pros/. Accessed June 20, 2018.

[14] Detmar SB Muller MJ Schornagel JH Wever LDV Aaronson NK: Health-related quality-of-life assessments and patient-physician communication: A randomized controlled trial. JAMA 2002;288:3027-3034. 12479768 10.1001/jama.288.23.3027

[15] Basch E: Patient-reported outcomes—Harnessing patients' voices to improve clinical care. N Engl J Med 2017;376:105-108. 28076708 10.1056/NEJMp1611252

[16] Lavallee D Chenok KE Love RM: Incorporating patient-reported outcomes into health care to engage patients and enhance care. Health Aff 2016;35:575-582. 10.1377/hlthaff.2015.1362 27044954

[17] Porter ME Larsson S Lee TH: Standardizing patient outcomes measurement. N Engl J Med 2016;374-376. 10.1056/NEJMp1511701 26863351

[18] Aaronson N Elliott T Greenhalgh J: User's Guide to Implementing Patient-Reported Outcomes Assessment in Clinical Practice. International Society for Quality of Life Research, 2011 http://www.isoqol.org/UserFiles/file/UsersGuide.pdf. 10.1007/s11136-011-0054-x 22048932

[19] Fung CH Hays RD: Prospects and challenges in using patient-reported outcomes in clinical practice. Qual Life Res 2008;17:1297-1302. 18709564 10.1007/s11136-008-9379-5 PMC3119524

[20] Snyder CF Herman JM White SM: When using patient-reported outcomes in clinical practice, the measure matters: A randomized controlled trial. J Oncol Pract 2014;10:e299-e306. 24986113 10.1200/JOP.2014.001413 PMC4161731

[21] Snyder CF Aaronson NK Choucair AK: Implementing patient-reported outcomes assessment in clinical practice: A review of the options and considerations. Qual Life Res 2012;21:1305-1314. 22048932 10.1007/s11136-011-0054-x

[22] Gerhardt WE Mara CA Kudel I: Systemwide implementation of patient-reported outcomes in routine clinical care at a children's hospital. Jt Comm J Qual Patient Saf 2018;44:441-453. 30071964 10.1016/j.jcjq.2018.01.002

[23] Lavallee D Austin E Franklin PD: How can health systems advance patient-reported outcome measurement? Jt Comm J Qual Patient Saf 2018;44:439-440. 30071963 10.1016/j.jcjq.2018.05.005 PMC7047893

[24] Biber J Ose D Reese J: Patient reported outcomes—Experiences with implementation in a University Health Care setting. J Patient Rep Outcomes 2018;2:34. 30175316 10.1186/s41687-018-0059-0 PMC6097980

[25] Burwell SM: Setting value-based payment goals-HHS efforts to improve U.S. health care. N Engl J Med 2015;372:897-899. 25622024 10.1056/NEJMp1500445

[26] Gensheimer SG Wu AW Snyder CF; PRO-EHR Users' Guide Steering Group; PRO-EHR Users' Guide Working Group: Oh, the places we'll go: Patient-reported outcomes and electronic health records. Patient 2018;11:591-598. 29968179 10.1007/s40271-018-0321-9

[27] Patient-Centered Outcomes Research Institute: User's Guide to Integrating Patient-Reported Outcomes in Electronic Health Records: Version: May 2017. www.pcori.org/sites/default/files/PCORI-JHU-Users-Guide-To-Integrating-Patient-Reported-Outcomes-in-Electronic-Health-Records.pdf.

[28] Aaronson N Elliott T Greenhalgh J; International Society for Quality of Life Research: User's Guide to Implementing Patient-Reported Outcomes Assessment in Clinical Practice, Version: January 2015. http://www.isoqol.org/UserFiles/2015UsersGuide-Version2.pdf.

[29] Carman KL Paez K Stephens J: Improving Care Delivery Through Lean: Implementation Case Studies. Rockville, MD, Agency for Healthcare Research and Quality, 2014.

[30] Rotenstein LS Agarwal A O'Neil K: Implementing patient-reported outcome surveys as part of routine care: Lessons from an academic radiation oncology department. J Am Med Inform Assoc 2017;24:964-968. 28339771 10.1093/jamia/ocx009 PMC7651917

[31] Nordan L Blanchfield L Niazi S: Implementing electronic patient-reported outcomes measurements: Challenges and success factors. BMJ Qual Saf 2018;27:852-856. 10.1136/bmjqs-2018-008426 30021802

[32] Zhang R Burgess ER Reddy MC: Provider perspectives on the integration of patient-reported outcomes in an electronic health record. JAMIA Open 2019;2:73-80. 30976756 10.1093/jamiaopen/ooz001 PMC6447042

